# Early Introduction of Novel and Less-Studied Food Allergens in the Plant-Based Era: Considerations for US and EU Infant Formula Regulations

**DOI:** 10.3390/nu15214530

**Published:** 2023-10-25

**Authors:** Carina Venter, Raanan Shamir, David Mark Fleischer

**Affiliations:** 1 University of Colorado/Children’s Hospital Colorado: Section of Pediatric Allergy and Clinical Immunology, Aurora, CO 80045, USA; david.fleischer@childrenscolorado.org; 2 Institute for Gastroenterology, Nutrition and Liver Disease, Schneider Children’s Medical Center of Israel, Faculty of Medicine, Tel Aviv University, Tel Aviv 74071-12-20, Israel; raanan@shamirmd.com

**Keywords:** plant based, nutrition, food allergy, infant growth

## Abstract

Early life feeding practices may affect the long-term health of individuals, particularly in terms of the development of non-communicable diseases, such as metabolic and allergic diseases. Accumulating evidence suggests that the interplay of breastfeeding and/or formula feeding followed by the introduction of solids plays a role in the occurrence of non-communicable diseases both in the short and long term. International food allergy guidelines recommend that breastfeeding women do not need to avoid food allergens and do not recommend any infant formula for allergy prevention. Guidelines regarding solid food introduction for food allergy prevention recommend the introduction of well-cooked eggs and peanuts around 4–6 months of age, and not to delay the introduction of other food allergens. There is also an increasing trend to feed infants a plant-based or plant-forward diet and have access to infant formulas based on plant-based ingredients. The use of novel plant-based infant formulas raises a few questions reviewed in this paper: (1) Do fortified, plant-based infant formulas, compliant with US Food and Drug Administration (FDA) regulations and European Food Safety Authority (EFSA) (European) guidelines, support adequate infant growth? (2) Are plant-based infant formulas suitable for the management of cow’s milk allergy? (3) Does feeding with novel, plant-based infant formulas increase the risk of food allergies to the food allergens they contain? (4) Does feeding infants plant-based food allergens in early life increase the risk of allergic and severe allergic reactions? The review of the literature indicated that (1) plant-based formulas supplemented with amino acids and micronutrients to comply with FDA regulations and EFSA guidelines, evaluated in sufficiently powered growth studies, can support adequate growth in infants; (2) currently available plant-based infant formulas are suitable for the management of CMA; (3) an early introduction and continuous intake of food allergens are more likely to prevent food allergies than to increase their risk; and (4) an early introduction of food allergens in young infants is safe.

## 1. Introduction

Early life feeding practices may affect the long-term health of individuals, particularly in terms of the development of non-communicable diseases such as metabolic and allergic diseases [1]. Guidelines recommend that infants should be exclusively breastfed for the first six months of life [2]. For food allergy prevention, from four to six months of age, infants are introduced to solids, which will overlap with breast and/or formula feeding [3,4,5]. Solid food introduction also includes the introduction of food allergens. An adequate introduction of solid food while breastfeeding continues is important for nutritional and developmental reasons. The aim of feeding during the first year of life is to achieve eating a family diet by 12 months of age [6]. This means that at age one, in addition to breastfeeding, the child will consume a diet like what the family is eating [7].

There is no evidence that breastfeeding prevents food allergy [5]. Nevertheless, for many other health and non-health reasons, breastfeeding is the recommended method of infant feeding, and the method of feeding recommended for infants at high risk for developing allergic diseases. International food allergy guidelines recommend that breastfeeding women do not need to avoid food allergens, and there is currently no infant formula that is recommended for food allergy prevention [4,8,9].

Guidelines for introducing solid foods for food allergy prevention have changed over time [10]. Current guidance advocates for the introduction of peanuts and well-cooked eggs around 4–6 months of age, as these are the only two food allergens that have been studied with clear outcomes. They also indicate that infants should only commence solid food introduction when they are developmentally ready to consume solid foods. The guidelines also advise not to delay the introduction of other food allergens. Finally, the guidelines suggest that diet diversity in early life may prevent the development of food allergies in later childhood [4,8,9]. Foods targeted for allergy prevention may be country-specific and should take cultural eating habits into account [4,8,9]. The introduction of these allergens to exclusively breastfed infants does not harm breastfeeding when properly conducted [11].

Another significant change seen in early life feeding practices is the increasing demand of parents to feed their infants a plant-based diet [7]. Physicians and other clinicians are progressively seeing families where parents choose to feed their infants and young children plant-based “milk” alternatives to cow’s milk for various reasons, such as medical conditions or cultural dietary preferences, or health-related perceptions such as a possible beneficial effect on the gut microbiome [12]. A plant-based diet focuses on the consumption of vegetables, fruits, legumes, and grains, but does not necessarily eliminate all animal-based foods. This is different from a vegan diet, which cuts out all animal-based products, and a vegetarian diet, which removes certain animal-based products. In adults, plant-based diets have been associated with clear benefits in health outcomes, such as the prevention of chronic diseases and improvement in health in those suffering from certain chronic diseases [13]. In children, current data are not that conclusive. As there are no data regarding plant-based diets, we here summarize data regarding vegan and vegetarian diets.

A systematic review on vegetarian diets indicated that due to the heterogeneity and small sample sizes of studies, it is difficult to draw firm conclusions on either the health benefits or risks of plant-based diets in children [14]. A position statement from the Academy of Nutrition and Dietetics (US) concludes that vegan and vegetarian diets are nutritionally acceptable and may prevent or treat many diseases. This position statement states further that vegetarian and vegan diets are appropriate for all ages and that plant-based diets are more environmentally sustainable than other diets [15]. However, as infants on plant-based diets may be at risk of nutritional deficiencies, it is recommended that these infants, if not breastfed, consume a suitably fortified plant-based formula based on rice and/or soy [16]. It is important to distinguish between plant-based formulas which have been supplemented appropriately to meet FDA (US) regulations and EFSA (European) guidelines [17] and plant-based beverages that are commercially available but not approved by the FDA and EFSA. These plant-based beverages may be supplemented with calcium, vitamin D, and several other nutrients [18]. To date, however, they are not supplemented with amino acids, so their nutrient composition does not comply with FDA standards for infant formula [19]. FDA and EFSA also recommend that infant formula should be supplemented with vitamins and minerals, as well as omega 3 (DHA) and omega 6 (ARA). Thus, these plant-based beverages are often nutritionally inadequate to support normal growth and development compared to toddler formulas or cow’s milk. Inappropriate use of plant-based beverages/milks has been associated with inadequate weight gain and growth, electrolyte imbalance, and inadequate nutrient intake. It is, however, difficult to determine if this is a direct association or an indirect association with cow’s milk avoidance [20,21,22,23].

The use of novel plant-based formulas is increasing, but raises the following concerns that need to be addressed:

(1) Do fortified plant-based infant formulas, compliant with FDA regulations and EFSA (European) guidelines, support adequate infant growth?
(2) Are plant-based infant formulas suitable for the management of cow’s milk allergy (CMA)?
(3) Does feeding with novel plant-based infant formulas increase the risk of food allergies to the food allergens they contain?
(4) Does feeding infants plant-based food allergens in early life increase the risk of allergic and severe allergic reactions?

This review paper will address the listed questions with an emphasis on a novel plant-based formula containing almonds and buckwheat.

## 2. Methods

For this narrative review, we performed a systematic search of the literature using six electronic databases (MEDLINE, EMBASE, CINAHL, Web of Science, Cochrane Library, and Scopus) to collect papers written in English. We used the search terms almond, tree nut, buckwheat, prevalence, incidence, sensitization, food allergy, oral food challenge, reported food allergy, specific IgE, and skin prick test. For prevalence data, available data from across the whole world were included, while guidelines and recommendations were limited to the US and Europe.

## 3. Overview

### 3.1. Do Fortified Plant-Based Infant Formulas, Compliant with FDA Regulations and European Food Safety Authority (European) Guidelines, Support Adequate Infant Growth? 

The American Academy of Pediatrics (AAP) highlights the importance of adequate growth and nutrition throughout childhood [24]. The North American Society for Pediatric Gastroenterology, Hepatology, and Nutrition (NASPGHAN) Nutrition Committee recently published a document [12] stating that that an inadequate nutritional intake can adversely affect a child’s nutritional status, growth, and development, and that cow’s milk intake plays a particularly important role in a child’s overall diet. The only currently available plant-based infant formulas that have been shown to support infant growth are hydrolyzed rice formula and soy formula containing intact proteins.

#### 3.1.1. Rice Formula

Rice hydrolysates can be either partially or extensively hydrolyzed. Although rice is rich in essential amino acids, it lacks some amino acids that are present in human milk. To ensure nutritional adequacy and growth in infants with CMA, hydrolyzed rice formulas are supplemented with lysine, threonine, and tryptophan, like other hypoallergenic formulas, and meet the micronutrient requirements for infant formulas [25]. In terms of concerns about the inorganic arsenic content of these formulas, recent reports reassure that arsenic content is within safe limits [26,27].

A study by Vandenplas et al. [28] showed that an extensively hydrolyzed rice formula supported normal weight gain within the first month of use and normalization of weight-for-age, weight-for-length, and BMI z-scores within a 6-month period. Several other studies have also shown that rice hydrolysates support growth in infants [29,30,31,32] (Table 1).

#### 3.1.2. Soy Formula

Soy formulas are supplemented with the amino acids methionine, taurine, and carnitine [33] and must comply with FDA guidance on micronutrient composition of infant formulas. Vandenplas et al. [34] performed a systematic review with a meta-analysis on the role of soy infant formulas in supporting growth and development. The authors identified fourteen randomized controlled trials which indicated that supplemental soy infant formulas support growth (weight and length gain) like human milk and cow’s milk-based infant formulas in infants. The AAP supports the use of soy-based infant formulas to support the normal growth and development of term infants who are not breastfed or fed with cow’s milk formula [35].

Conclusion

We conclude that the available evidence indicates that plant-based formulas, supplemented with the amino acids and micronutrients required to comply with FDA regulations and EFSA guidelines, sufficiently support growth in infants.

Recommendation

Plant-based formulas, supplemented with the amino acids and micronutrients required to comply with FDA regulations and developed based on sufficiently powered growth studies, can support normal growth in infants.

### 3.2. Are Plant-Based Infant Formulas Suitable for the Management of Cow’s Milk Allergy?

#### 3.2.1. Rice-Based Formula

A small number of trials concluded that no allergic reactions, commonly associated with CMA, were triggered by the consumption of hydrolyzed rice formula in patients with CMA [28,36,37,38,39]. Currently, no studies have been carried out to test if rice hydrolysates can be used in children with a rice-triggered food allergy. Formulas included extensively hydrolyzed [28,36,37] and partially hydrolyzed formulas [38], and one formula where the level of hydrolysis was unclear [39].

#### 3.2.2. Soy-Based Formula

Based on a review of three studies [40,41,42], it was estimated that 10–14% of infants with CMA may also develop an allergy to soy-based formula, in which case the use of soy formula was not recommended [43]. However, recent estimates of soy allergy are much lower, and current recommendations do find a place for the use of soy-based formulas in children with CMA [44].

### 3.3. Does Feeding Novel Plant-Based Infant Formulas Increase the Risk of Food Allergies to the Food Allergens They Contain?

In this review, we focus on the prevalence of two allergens incorporated into a novel almond-milk-based formula (almond and buckwheat) which is currently being used in the US and the EU. This plant-based formula is undergoing studies for its ability to support adequate infant growth and its suitability for use in infants with CMA. Understanding the prevalence of allergies to these food allergens provides information on the potential size of the problem, i.e., when the prevalence of a food allergy is very low, the potential risk of increasing the prevalence of the food allergy is also low. This review will focus on our current understanding of almond and buckwheat allergies worldwide. We will also further investigate if an early and continuous introduction of food allergens in early life, which is the case for feeding with infant formula, changes the risk of developing an allergy to the food allergen being fed.

#### 3.3.1. Prevalence of Almond Allergy

Almonds are a fruit belonging to the Rosaceae family. Several allergens have been isolated from almonds, including Pru du 1 (2S albumin, spare protein), Pru du 2 (conglutin, 7S globulin, reserve protein), Pru du 3 (LPT, lipid transporting protein), Pru du 4 (profilin), Pru du 5 (ribosomal protein P2), and Pru du 6 (amandine–hexameric protein of 360 kDa 11S legumin). The main allergen in almonds, 2S albumin, may lead to cross-sensitization to walnuts, sunflower seeds, and peanuts, though clinically significant cross-reactions due to primary food allergies are rarely encountered [45]. Pollen food allergy syndrome is the occurrence of sensitization to pollen that contains similar allergens to a food, e.g., some people with birch pollen allergy may have itching in their mouth when they eat almonds, apples, carrots, hazelnuts, kiwis, peaches, pears, plums, cherries, or soy. Another example of pollen food allergy syndrome are the cross-reactions seen between grass pollen and celery, melons, oranges, peaches, and tomatoes. The main allergens responsible for these cross-reactions are PR-10 proteins and profilin. Clinical cross-reactions in patients with pollen food allergy syndrome have been reported, but these presentations of food-related symptoms are encountered in children old enough to have allergic rhinitis to pollen (usually 3–5 years of age or older) and have not been described in infants who are too young to be sensitized to pollens [46]. These reactions, however, are usually limited to the oropharynx and consist of localized pruritus, and they are extremely unlikely to lead to severe food allergic reactions.

The prevalence of population-based almond allergy has not been widely studied. Tree nut allergies include Brazil nuts, cashews, hazelnuts, pecans, pistachios, walnuts, and almonds. Tree nut allergy prevalence varies by age, region, and food allergy definition, and ranges from less than 1% to approximately 3% worldwide [47]. Studies from the EU report that hazelnuts are the most common source of tree nut allergy. In the UK, Brazil nuts are the most common source of tree nut allergy, primarily based on studies from the Isle of Wight, and cashews are the most common source of tree nut allergy in Australia. Recent US data from Gupta et al. [47] report almond (0.7%) and cashew (0.7%) allergies as the most common tree nut allergies in 0–17-year-old children, though still much lower than the 2.7% cashew nut allergy rate reported in 6-year-old Australian children. Almond allergy, including reported allergy and/or sensitization, has been studied in seven countries: Hungary [48], Iceland [49], Sweden [49], Turkey [50], the UK [51], US [52,53], and Australia [54]. (Table 2).

- Sensitization

Using skin prick tests (SPTs), Venter et al. [56] described a sensitization rate of 0.3% in 3-year-old children and Roberts et al. [57] found a sensitization rate of 0.5% in 7-year-old children in the UK. No child in Turkey aged 6–9 years was found to be sensitized to almonds [50]. Similarly, using specific IgE testing, no elderly (60–97 years) persons in Hungary were sensitized to almonds [48].

- Self-reported rates

Importantly, there were no self-reports of almond allergy in children aged 18 months in both Iceland and Sweden [49]. Another study from Sweden indicated a self-reported almond allergy rate of 3.8% in 4-year-old children [55].

- Self-reported clinician’s/doctor’s diagnosis (not based on oral food challenges)

Surveys from the US indicated self-reported doctor’s diagnoses of almond allergy in 0.9% of adults in 1999 [52] and 1.6% in 2010 [58]. More recently, Gupta et al. [53] indicated self-reports of doctor’s diagnoses of almond allergy in 0.2% of US infants and 0.7% of 0–17-year-old children. This should be seen in the context of peanut allergy, which occurs in 2.2% of children up to 17 years of age and 0.6% of infants up to 1 year of age. In US infants at one year of age, almond allergy (0.2%) is also much lower than CMA (1.5%) and soy allergy (0.4%) [53].

- Oral food challenges (OFCs).

Based on OFCs, the gold standard for food allergy diagnosis, there were no 6–9-year-old children in Turkey [50], nor any one- [51] or two- [56] year-old children in the UK diagnosed with an almond allergy. At 3 years of age [56], 0.2% of UK-based children were diagnosed with an almond allergy, but none at age one year.

Conclusion

Very little data regarding true food allergy to almonds are available. Only one study reported OFC-proven almond allergy in infants; that study found no infants to be almond-allergic, but 0.2% of these children were almond-allergic by age 3 years.

Recommendation

More robust studies are required to estimate the true prevalence of almond allergy in infants.

#### 3.3.2. Prevalence of Buckwheat Allergy

Buckwheat originates from China, and the two buckwheat species include common buckwheat (*Fagopyrum esculentum*) and Tartary buckwheat (*Fagopyrum tartaricum*). Food allergens in common buckwheat include Fag e1, Fag e2, and Flag e3, and those in Tartary buckwheat include Flag t1, Fag t2, and Fag t3. Clinical cross-reactivity has been described with peanuts, latex, coconut, quinoa, and poppy seeds, but is rare [59] (Table 3).

- Self-reports

Self-reported prevalence figures are available from Korea [60,61], Japan [62], and China [63], but no studies have implemented OFCs to confirm the self-reported numbers. In one Korean study [60], 0.1% of 12–15-year olds and 6–12-year olds reported a buckwheat allergy. Another study reported that 0.3% of 6–16-year-olds consider themselves to have a buckwheat allergy [61]. In Japan, 0.22% of 5–10-year-olds reported a buckwheat allergy, [62] and in China, 0.4% of 13–21-year-olds reported a buckwheat allergy [63]. Recently, a case of a 4-year-old with food protein-induced enterocolitis (FPIES) triggered by buckwheat was reported in Japan based on an OFC, but FPIES triggered by buckwheat has not been reported in infants [64].

No data are available regarding sensitization to buckwheat, self-reported clinical diagnosis, or clinical diagnosis based on a good clinical history supported by sensitization or an oral food challenge.

Conclusion

There are no OFC studies to estimate the prevalence of buckwheat allergy, and there is a dearth of information on the prevalence of buckwheat allergy based on self-reported prevalence. There are no prevalence data for children and infants from non-Asian countries of origin. It is currently not possible to provide a true estimate of buckwheat allergy.

Recommendations

Robust studies are required to estimate the true prevalence of buckwheat allergy.

#### 3.3.3. Early Introduction of Food Allergens

An early or timely introduction of food allergens, such as peanuts and eggs, is recommended by all international food allergy prevention guidelines, alongside no delay in the introduction of other foods or food allergens once the infant is ready to commence solid foods [4,8,9]. Many food allergens, such as milk, eggs, wheat, cod, peanuts, sesame, buckwheat, almonds, cashews, shrimp, walnuts, salmon, hazelnuts, or a combination of these, have been studied in early introduction trials (Table 4).

However, the age of introduction of almond and buckwheat for the prevention of these food allergens has not been studied. Thirteen well-conducted studies have been performed looking at the early introduction of food allergens and food allergy outcomes. Some of these, however, are cohort studies rather than randomized blinded controlled trials. Of note are the seven studies [11,72,73,74,75,76,77] focusing on the introduction of cow’s milk. The age of introduction varied between birth and up to 3–4 months of age. Some of these studies indicate that early introduction of milk, as early as at birth, followed by consistent intake of cow’s milk reduces the prevalence of CMA. None of the other food allergens have been introduced this early.

A recent systematic review [78] concluded that an earlier introduction of multiple allergenic foods during infancy was associated with a lower risk of developing food allergies. The timing of introducing cow’s milk and the risk of developing CMA showed a very low level of evidence or certainty. However, sensitivity analyses restricted to low-risk-of-bias data [74,77] reduced heterogeneity and showed a significant risk reduction in the case of early and continuous introduction of cow’s milk (RR, 0.32; 95%CI, 0.09–1.18; I2 = 0%). One of these studies, by Nishimura et al. [77], used a multi-allergen mix which included buckwheat. The overall incidence of food allergy by 18 months of age was significantly lower in the multi-allergen mix than in the placebo group (risk ratio 0.301 [95% CI 0.116–0.784]; *p* = 0.0066). None of the infants in either the multi-allergen mix or placebo group developed a buckwheat allergy. Another multi-allergen mix study [76] included almonds, and as with buckwheat, assessment for individual almond allergy was not conducted. However, the percentage of participants able to consume 8 g of total protein during the OFC was significantly higher in the group using the mixed protein supplement containing almond compared to the controls (*p* < 0.01). These two studies [76,77], alongside the EAT study [11], could be considered as studies investigating the role of food allergen diversity on food allergy outcomes. Overall, there is evidence to suggest that food allergen diversity by 12 months of age has been significantly associated with reduced odds of food allergy by 10 years of age [79].

A recent observational study in Japan indicated that buckwheat was avoided by 71.9% of families for infants at 18 months of age, but this avoidance or consumption was not associated with any allergic outcomes [80]. Another study from Japan investigated predictors for diagnosing buckwheat allergy [81] and found a higher buckwheat sIgE/total IgE was a significant predictor for a positive OFC. The age of introduction of buckwheat was, however, not reported.

Conclusion

There are currently no studies investigating the age of introduction of almonds or buckwheat as single allergens that may give an indication if an early introduction of these allergens may increase or reduce the risk of food allergies. However, studies focusing on single foods such as milk, egg, or peanut, or those using food allergen mixes, indicate that an early introduction and consistent intake of these foods (allergens) lead to a reduction in these specific food allergies or overall food allergy prevalence. Doses varied greatly in these studies, which may indicate that timing and frequency may be more important than the dose consumed.

Recommendation

Early introduction and continuous intake of food allergens are more likely to prevent food allergies than to increase their risk. The most beneficial dose of food allergens for tolerance induction is unclear.

### 3.4. Does Feeding Infants Plant-Based Food Allergens in Early Life Increase the Risk of Allergic and Severe Allergic Reactions?

The incidence of severe allergic reactions to buckwheat, including anaphylaxis, can be estimated as 0.1–0.01 cases per 100,000 person-years [59]. The incidence of almond anaphylaxis is not clear [82,83].

It is estimated that the risk of a severe reaction occurring in infants in the general population upon first ingestion is less than 0.08% [84]. Additionally, even if anaphylaxis were to occur at first ingestion, recent evidence shows that infant anaphylaxis is milder than that in older children [85,86]. There have been no reported fatalities due to early introduction, and it is generally presumed that infants do not have anaphylaxis, although it can occur. The food allergy prevention guidance from the British Society for Allergy and Clinical Immunology (BSACI) states that “no life-threatening reactions to date have been reported as a result of earlier introduction of potential allergens into the infant diet” [87]. In the LEAP study, all infants challenged to peanut at recruitment had mild reactions, and none of the infants had anaphylaxis or required adrenaline [66]. The only studies reporting severe symptoms in infants used raw/pasteurized egg powder; the STAR study was stopped as one-third of the subjects experienced an allergic reaction, which included anaphylaxis, when fed the raw egg product [67]. Similarly, in the HEAP study, 10 of 23 children were excluded from the study as they developed anaphylaxis during the entry OFC [71]. These concerns were not raised in other early introduction trials.

Conclusion

The available data suggest that early introduction of food allergens, other than the introduction of raw eggs, is highly unlikely to cause a severe allergic reaction in young infants, with the risk of a severe reaction occurring below 0.1%. Furthermore, there have been no severe reactions reported to almonds or buckwheat in infants. Early introduction of food allergens other than raw egg in young infants is safe.

Recommendation

We recommend that food allergens can be safely introduced into the diets of young infants.

## 4. Overall Conclusions

The use of novel plant-based formulas is increasing. A formula based on almond and buckwheat proteins, fortified with amino acids to ensure the presence of all essential amino acids, vitamins, and minerals as well as omega 3 (DHA) and omega 6 (ARA), may be a suitable option for families requiring a plant-based option. This formula is currently undergoing studies to show that it supports normal growth in infants and children and is suitable for the treatment of CMA. It is unclear what the true prevalence of almond and buckwheat allergies is, if an early introduction of almond and buckwheat may increase the risk of developing allergies to these foods or decrease this risk, and if early introduction may lead to severe reactions in infants. However, our review of the literature indicates that (1) plant-based formulas supplemented with amino acids and micronutrients to comply with FDA regulations and EFSA guidelines, evaluated in sufficiently powered growth studies, can support adequate growth in infants; (2) currently available plant-based infant formulas are suitable for the management of CMA; (3) an early introduction and continuous intake of food allergens are more likely to prevent food allergies than increase their risk; and (4) early introduction of food allergens in young infants is safe.

## Figures and Tables

**Table 1:** Summary of prospective clinical studies using hydrolyzed rice formula.

Study	Number of Study Participants	Age of Participants	Study Duration	Classification of Infants Included in the Study	Number of Study Participants Receiving Rice Hydrolysate	Number of Study Participants Receiving Other Formulas	Number of Participants in Control Groups
Randomized [29]	16	6–16 months	6 months	CMA	8	8 soy	
Randomized [30]	80	2 days	4 months	Healthy	32	33 cow’s milk	
Randomized [31]	160	5 months	6 months	CMA	30	31 extensively hydrolyzed 32 soy	32 breastfed
Non-randomized [32]	88	29–25 months	2 years	CMA	15	26 extensively hydrolyzed 17 soy	30 healthy
No control [28]	40	<6 months	6 months	CMA	40	NA	

Legend: This table provides an overview of studies conducted on infants using rice hydrolysates. All these studies reported that the rice hydrolysates supported normal growth in study participants. Abbreviations: CMA: cow’s milk allergy; NA—no data available.

**Table 2:** Prevalence of almond sensitization, self-reported symptoms, self-reported clinical diagnosis, or clinical diagnosis of almond allergy based on good clinical history supported by sensitization or oral food challenge.

Country	Age of Study Participants	Sensitization—SPT (% with a Positive SPT)	Sensitization—IgE (% with Positive Specific IgE Levels)	Self-Reported Reactions	Self-Reported Reactions Based on a Clinical Diagnosis	Sensitization (Positive SPT or Specific IgE Levels and Good Clinical History of Allergic Reaction)	Positive OFC
Hungary [48]	20–69 years	NA	NA	NA	NA	NA	NA
Hungary [48]	60–97 years	NA	0 (0–0.42)	NA	NA	NA	NA
Iceland [49]	18 months	NA	NA	0 (0–1.5)	NA	0 (0–1.5)	NA
Sweden [49]	18 months	NA	NA	0 (0–1.4)	NA	0 (0–1.4)	NA
Sweden [55]	4 years	NA	NA	3.8 (3.1–4.7)	NA	NA	NA
Turkey [50]	6–9 years	0	NA	0	NA	NA	0
United Kingdom [51]	1 year	NA	NA	NA	NA	NA	0 (0–0.5) *n* = 900
United Kingdom [56]	2 years	NA	NA	NA	NA	NA	0 (0–0.6)
United Kingdom [56]	3 years	0.3 (0.0–1.2)	NA	NA	NA	NA	0.2 (0.0–0.9)
United Kingdom [57]	7 years	0.5 (0.2–0.9)	NA	NA	NA	NA	
United States [52]	>18 years	NA	NA	NA	0.9 (0.7–1.1)	NA	NA
United States [58]	≥18 years	NA	NA	NA	1.6 (1.4–1.9)	NA	NA
United States [53]	All children < 18 years	NA	NA	NA	0.7 (0.6–0.8)	NA	NA
United States [53]	Infants < 1 year	NA	NA	NA	0.2 (0.1–0.5)	NA	NA
Australia [54]	6 years	NA	NA	NA	0.3 (0.1–0.5)	NA	0.3 (0.1–0.5)

Table legend: This table provides a summary of all population-based studies reporting on almond sensitization, self-reported symptoms, self-reported clinical diagnosis, or clinical diagnosis of almond allergy based on good clinical history supported by sensitization or oral food challenge. Abbreviations: NA—no data available; OFC—oral food challenge; SPT—skin prick test.

**Table 3:** Prevalence of self-reported allergic reactions to buckwheat.

Country	Age of Study Participants	Sensitization—SPT (% with a Positive SPT)	Sensitization—IgE (% with Positive Specific IgE Levels)	Self-Reported Reactions	Self-Reported Reactions Based on a Clinical Diagnosis	Sensitization (Positive SPT or Specific IgE Levels and Good Clinical History of Allergic Reaction)	Positive OFC
Korea [60]	12–15 years	NA	NA	0.1 (0.1–0.2)	NA	NA	NA
Korea [60]	6–12 years	NA	NA	0.1 (0.1–0.1)	NA	NA	NA
Korea [61]	6–16 years	NA	NA	0.3	NA	NA	NA
Japan [62]	5–10 years	NA	NA	0.22	NA	NA	NA
China [63]	13–21 years	NA	NA	0.4	NA	NA	NA

Table legend: This table provides a summary of all population-based studies reporting self-reported symptoms to buckwheat. There are currently no studies available reporting on population-based sensitization, self-reported clinical diagnosis, or clinical diagnosis based on good clinical history supported by sensitization or oral food challenge. Abbreviations: NA—no data available; OFC—oral food challenge; SPT—skin prick test.

**Table 4:** Summary of randomized controlled trials of early allergenic food introduction for food allergy prevention adapted from [65].

Study Acronym	Full Study Title	Study Type	Population Included	Food Allergen Introduction Intervention	Primary Outcome	Results
LEAP (UK) [66]	Learning Early About Peanut Allergy	Non-blinded RCT (*n* = 640)	High-risk infants with moderate to severe eczema and/or egg allergy	Thrice-weekly consumption of 2 g of peanut protein vs. complete avoidance of peanut after randomization at 4–11 months, through 60 months of life	IgE-mediated peanut allergy based on OFC at month 60	ITT analysis showed prevalence of peanut allergy of 13.7% in the avoidance group vs. 1.9% in the consumption group (*p* < 0.001)
STAR (Australia) [67]	Solids Timing for Allergy Reduction	Blinded RPCT (*n* = 86)	High-risk infants with moderate to severe eczema	Daily consumption of 0.9 g raw whole egg powder (0.4 g protein/day) vs. placebo powder from 4 to 8 months of age Cooked egg consumption from 8 months onwards	IgE-mediated egg allergy at 12 months based on positive SPT and egg OFC	Study was terminated early as 1/3 of patients reacted to egg during entry OFC At 12 months, 33% had egg allergy in egg consumption group vs. 51% in control (not significant)
PETIT (Japan) [68]	Preventing egg allergy in infants with AD	Blinded RCT (*n* = 121)	High-risk infants with atopic dermatitis	Daily consumption of 50 mg heated egg from 6 to 9 months Daily consumption of 250 mg heated egg from 9 to 12 months	IgE-mediated egg allergy at 12 months of age based on OFC	Prevalence of egg allergy was 37.7% in placebo vs. 8.3% in egg consumption group (*p* = 0.0013) No SAEs were reported
STEP (Australia) [69]	Starting Time for Egg Protein	Blinded RPCT (*n* = 820)	Intermediate risk: atopic mothers (allergic disease + positive envir SPT Infants: no allergic diagnosis	Daily consumption of 0.9 g raw whole egg powder (0.4 g protein/day) vs. placebo powder from 4 to 6.5 months	IgE-mediated egg allergy at 12 months based on positive SPT and egg OFC	No significant differences in egg allergy between the groups No anaphylactic reactions were reported during initial egg introduction
BEAT (Australia) [70]	Beating Egg Allergy Trial	Blinded RPCT (*n* = 319)	Intermediate risk: infants with 1st degree relative with atopy Infants: neg egg SPT	Daily consumption of 350 mg raw, whole egg protein vs. placebo powder from 4 months of age Cooked egg from 8 months of age	Sensitization to egg by SPT at 12 months of age	Subjects in egg consumption group had significantly less egg sensitization (10.7% vs. 20.5%, *p* = 0.03) than the placebo group No anaphylactic reactions were reported during initial egg introduction
HEAP (Germany) [71]	Hens Egg Allergy Prevention	Blinded RPCT (*n* = 406)	Normal-risk general population Infants with IgE < 0.35 kU/L at enrollment	Thrice-weekly consumption of 2.5 g egg protein from 4 to 6 months of age until 12 months	Sensitization to egg based on egg IgE ≥ 0.35 kU/L at 12 months of age	No significant different in egg allergy between the egg consumption (2.1%) and the placebo (0.6%) groups High rate of anaphylaxis during egg introduction at entry experienced
ABC Trial (Japan) [72]	Atopy Induced by Breastfeeding or Cow’s Milk Formula	Non-blinded RCT (*n* = 312)	Intermediate risk: infants with 1st degree relative with atopy	Subjects were randomized to 2 interventions: (1) Active group: breast milk (BF) and/or cow’s milk (CMF) in first 3 days of life (2) Placebo group: BF and/or amino acid (EF)-based formula in first 3 days	Primary outcome: Sensitization to cow’s milk at 24 months Secondary outcomes: IgE-mediated milk allergy at 24 months based on OFC	Sensitization and cow’s milk allergy lower in the BF/EF group vs. the BF plus CMF group (relative risk 0.52 [sensitization] and 0.2 [cow’s milk allergy])
SPADE (Japan) [73]	Strategy for Prevention of Milk Allergy by Daily Ingestion of Infant Formula in Early Infancy	Non-blinded RCT (*n* = 518)	Normal-risk general population	Subjects were randomized between 1 and 2 months of age to daily consumption of at least 10 mL of cow’s milk formula (CMF) or avoidance of CM (soy formula) Ongoing breastfeeding recommended until 6 months of age	IgE-mediated milk allergy at 6 months of age based on OFC	ITT analysis showed that 2/242 in the ingestion-group (0.8%) vs. 17/249 in the avoidance group (6.8%) had milk allergy (*p* < 0.001) No SAEs were reported during the screening OFC
EAT (UK) [11]	Enquiring About Tolerance	Non-blinded RCT (*n* = 1303)	Normal risk: exclusively breastfed until allergenic foods introduced	The early introduction group (EIG) introduced 2 g of peanut, cooked egg, cow’s milk, sesame, whitefish, wheat protein twice weekly at 3 months The standard introduction group (SIG) followed UK guidance to start introduction of solid foods from 6 months of age	IgE-mediated food allergy to at least 1 of the 6 allergens at 1 or 3 years of age based on OFC	ITT analysis showed no difference in food allergy between EIG (5.6%) vs. SIG (6.1%) (*p* = 0.32) PP analysis showed significantly less prevalence of peanut allergy (*p* = 0.003) and egg allergy (*p* = 0.009) in EIG vs. SIG
PreventADALL (Norway, Sweden) [74]	Preventing Atopic Dermatitis and ALLergies	Non-blindedRCT (*n* = 2397)	Normal-risk general population	Randomized to 4 intervention groups: (1) no intervention; (2) skin; (3) food; (4) combined skin and food. Food: introduction of peanut, cow’s milk, egg, and wheat from 3 months of age vs. no specific advice on food intro	IgE-mediated peanut, cow’s milk, egg, or wheat allergy at 36 months of age based of OFC	ITT analysis showed prevalence of food allergy reduced in food intervention group compared to no food intervention group: risk difference −1.6% [95% CI −2.7 to −0.5]; OR 0.4, [95% CI 0.2 to 0.8] Reduced risk of food allergy was seen with peanut but not with other 3 foods
COMEET Israel [75]	Early, continuing exposure to cow’s milk formula and cow’s milk allergy	Allocated based on parental preference of feeding (*n* = 1992)	Normal-risk general population	Study subjects were allocated to either (1) exclusive breastfeeding (EBF) or (2) at least 1 feed with CMF (+/− breastfeeding) daily for the first 2 months of life	IgE-mediated cow’s milk allergy by 12 months of age CMA was defined as having at least 2 of 3 criteria: (1) positive OFC (2) sensitization to cow’s milk (3) clear history of immediate reactions	IgE-mediated CMA was 1.58% in the EBF group vs. 0 in “mixed feeding” group (*p* < 0.001) IgE-mediated CMA was 0.7% in the per-protocol EBF group vs. 3.27% in breastfed infants with irregular cow’s milk exposure in first 2 months
Japan [7]	Early introduction of very small amounts of multiple foods to infants	Blinded RCT (*n* = 163)	High-risk infants with atopic dermatitis	Study subjects were randomized to either (1) mixed-food-allergen powder which contained egg, milk, wheat, soybean, buckwheat, and peanuts, or (2) placebo powder at 3–4 months. The mixed-allergen powder contained 2.5 mg, 7.5 mg, and 20 mg of each food allergen plus probiotics—infants increased doses every 2 weeks	IgE-mediated food allergies up to 18 months Food allergy was defined as - sensitized to the food plus - clear history of reactions and/or a positive OFC	Food allergy was significantly lower in the mixed-powder group by 18 months vs. the placebo group (7/83 vs. 19/80, *p* = 0.0066)
US [76]	Early Introduction of Multi-Allergen Mixture for Prevention of Food Allergy: Pilot Study	Non-blinded RCT (*n* = 180)	General population	Infants were randomized to eat (1) single-food-allergen powder (milk, egg, or peanut at 300 mg protein per day: 2100 mg protein/food allergen/week) vs. (2) two-food-allergen powder (milk/egg, egg/peanut, milk/peanut at 300 mg per mix per day: 1050 mg protein/food allergen/week) vs. (3) a multiple-food-allergen mix of 10 different food allergens (milk/egg/peanut/cashew/almond/shrimp/walnut/wheat/salmon/hazelnut)ba at 3.1) low [300 mg per day: 21 (3 mg × 7) mg/food allergen/week], 3.2) medium [63 (9 mg × 7) mg/food allergen/week], or 3.3) high doses [210 (30 mg × 7) mg/food allergen/week]) vs. no early introduction at 4–6 months	IgE-mediated food allergy at 2 and 4 years using OFC	Study participants in all three mixes of the 10 food allergens, irrespective of the dose, were significantly more likely to consume 8 g food allergen protein than the other groups (q < 0.01)

Legend: This table provides a summary of current studies which conducted studies on early introduction of food allergens, giving an overview of study methodology and outcomes. Abbreviations: OFC, oral food challenge; SPT, skin prick test; RCT, randomized controlled trial; RPCT, randomized, placebo-controlled trial; ITT, intention-to-treat; CMA, cow’s milk allergy; AD, atopic dermatitis; SAE, serious adverse event.

## Data Availability

Not applicable.
